# Habitat and Forage Associations of a Naturally Colonising Insect Pollinator, the Tree Bumblebee *Bombus hypnorum*

**DOI:** 10.1371/journal.pone.0107568

**Published:** 2014-09-26

**Authors:** Liam P. Crowther, Pierre-Louis Hein, Andrew F. G. Bourke

**Affiliations:** 1 School of Biological Sciences, University of East Anglia, Norwich Research Park, Norwich, Norfolk, United Kingdom; 2 IUT Nancy-Brabois, Rue du Doyen Urion, Villers-les-Nancy CS, France; University of Sydney, Australia

## Abstract

Bumblebees (*Bombus* species) are major pollinators of commercial crops and wildflowers but factors affecting their abundance, including causes of recent population declines, remain unclear. Investigating the ecology of species with expanding ranges provides a potentially powerful means of elucidating these factors. Such species may also bring novel pollination services to their new ranges. We therefore investigated landscape-scale habitat use and foraging preferences of the Tree Bumblebee, *B. hypnorum*, a recent natural colonist that has rapidly expanded its range in the UK over the past decade. Counts of *B. hypnorum* and six other *Bombus* species were made in March-June 2012 within a mixed landscape in south-eastern Norfolk, UK. The extent of different landscape elements around each transect was quantified at three scales (250 m, 500 m and 1500 m). We then identified the landscape elements that best predicted the density of *B. hypnorum* and other *Bombus* species. At the best fitting scale (250 m), *B. hypnorum* density was significantly positively associated with extent of both urban and woodland cover and significantly negatively associated with extent of oilseed rape cover. This combination of landscape predictors was unique to *B. hypnorum*. Urban and woodland cover were associated with *B. hypnorum* density at three and two, respectively, of the three scales studied. Relative to other *Bombus* species, *B. hypnorum* exhibited a significantly higher foraging preference for two flowering trees, *Crataegus monogyna* and *Prunus spinosa*, and significantly lower preferences for *Brassica napus*, *Glechoma hederacea* and *Lamium album*. Our study provides novel, quantitative support for an association of *B. hypnorum* with urban and woodland landscape elements. Range expansion in *B. hypnorum* appears to depend, on exploitation of widespread habitats underutilised by native *Bombus* species, suggesting *B. hypnorum* will readily co-exist with these species. These findings suggest that management could target bumblebee species with distinctive habitat requirements to help maintain pollination services.

## Introduction

There is abundant evidence that bees and other insect pollinators are in global decline [1], [2], [3], [4]. Because 9.5% of global food production is attributable to wild insect pollination [5], pollinator losses threaten food security. Furthermore, such losses cannot be entirely mitigated by use of managed populations of honey bees (*Apis*), as these have been shown to complement but not replace the pollination services provided by wild pollinators across diverse agricultural systems [6].

In common with congeneric species elsewhere in the world, many bumblebee (*Bombus*) species in the UK have undergone declines over recent decades. Of the 20 recorded social species, three have gone nationally extinct and all but six are considered to have undergone range contractions [7]. Across species, the extent of these declines has been greater in species with later-starting colony cycles (i.e. with later nest foundation, build-up of worker numbers and production of sexuals) and smaller global ranges [7]. Because of the habitat associations of such species, this means that declines have occurred disproportionately in species that tend to be more dependent on specific habitats (e.g. unimproved grassland), which have been greatly reduced in extent [8]. In effect, the *Bombus* species that remain common and widespread tend to be generalists that can survive in a typical landscape mosaic of farmland, urbanised areas and isolated patches of semi-natural habitat, whereas those that have declined are restricted to fragmented patches of high-quality habitat. Since historical records indicate that many of these now-rare species were once widespread, structural changes have clearly altered the agricultural mosaic to reduce its suitability for many species [8]. In addition, it should be noted that, although widespread species are still extant in much of their historical ranges, there is insufficient evidence to conclude they have not also undergone declines in abundance.

The structural changes to agricultural habitats associated with bumblebee declines are reductions in floristic diversity, quantity of forage and abundance of nesting sites [9], [10]. Agriculturally-managed grasslands have lower floristic diversity and density largely through heavy use of synthetic nitrogen and more frequent and earlier cutting regimes [11]. Therefore the bumblebee species that persist in the altered agricultural environment rely extensively on wild flowers in the margins of agricultural fields with briefly available peaks of forage from mass flowering crops [12]. Although mass flowering crops such as oilseed rape (*Brassica napus*) are being cultivated on larger scales, benefits to *Bombus* populations may be limited by the early timing and brevity of their flowering period relative to *Bombus* colony cycles [13].

Understanding the reasons behind range expansions of *Bombus* species may provide clues as to the factors that make the difference between population decline and population stability in the genus as a whole. In addition, range-expanding species potentially add to the suite of pollination services provided by native pollinators. This study therefore focuses on a notable outlier to the trend for population decline among *Bombus* species, the Tree Bumblebee *Bombus hypnorum* (Linnaeus), which is currently undergoing a rapid range expansion in the UK. First recorded in southern England in 2001 [14], *B. hypnorum* now occurs throughout much of England, Wales and southern Scotland, and so has undergone a northwards range expansion of almost 600 km in 12 years [15]. Although the possibility of accidental or deliberate introduction cannot be excluded, *B. hypnorum* is most likely to be a natural colonist of the UK, since it is not used or traded as a commercial pollinator. It presumably reached southern England by autonomous dispersion across the English Channel from the closest neighbouring area of the pre-2001 range, northern France. *B. hypnorum* has a large global range, encompassing most of Europe and Asia, and occupies a wide range of biotopes [14]. In pristine landscapes, *B. hypnorum* is associated primarily with boreal forests at higher latitudes and montane forests at lower latitudes. Consistent with these associations, the presence of *B. hypnorum* is predicted by length of boreal forest edges in Estonian populations [16]. However, there is no clear indication of its habitat use in anthropogenic landscapes such as those exemplified by the agricultural mosaic in the UK.

*B. hypnorum* appears to have an early-starting colony cycle [17], which, along with a large global range, would place it among species resilient to decline according to the analysis of Williams [7]. In addition, observations suggest that, in non-boreal, lowland areas, *B. hypnorum* favours human settlements [18]. It is also suspected of exhibiting a facultatively bivoltine colony cycle [19] and, unlike most UK *Bombus* species, nests in above-ground cavities [17], including holes in trees and walls. An early-starting colony cycle, association with human settlement, facultative bivoltinism and above-ground nesting may all be contributing to the range expansion of *B. hypnorum*. Nonetheless, it remains unclear what factors underlie this rapid expansion on a land mass in which other *Bombus* species are declining and subject to pressures of a changing agricultural landscape. Moreover, recent work suggests that *B. hypnorum* has spread within the UK despite relatively high levels of parasite prevalence and low levels of genetic diversity [20]. In addition, although climate change could be contributing to the UK expansion of *B. hypnorum*, an expansion mirrored by some native species in the far north of the UK [21], it is unclear why some species but not others should be affected by climate change. Furthermore, existing data suggest that the main pattern of change in the ranges of declining *Bombus* species in Europe is one of contraction towards the climatic range centre, modulated by the ecological suitability of available habitat [22]. This implies that even if climate change is contributing to the range expansion of *B. hypnorum* in the UK, it remains important to establish the ecological determinants of the success of this species.

Overall, no previous study has quantified the landscape-scale habitat use and foraging preferences of *B. hypnorum* in its new range in the UK. We therefore aimed, within this population, (i) to quantify the relationship between the frequency of occurrence of *B. hypnorum* and landscape elements potentially providing nesting and foraging habitats, and (ii) to quantify the foraging preferences of *B. hypnorum* relative to those of other *Bombus* species.

## Materials and Methods

### Observations of Bees

The study was conducted in south-eastern Norfolk, UK, an area with landscapes typical of the mixed agricultural, semi-natural and urban landscapes found in southern England as a whole. *B. hypnorum* was first recorded in Norfolk in 2008 [23]. Forty-two sampling sites were selected at random from a set of 120 possible locations identified using an Ordnance Survey map as accessible using public rights of way, within an area of approximately 26×23 km. Post-selection inspection showed that they comprised a broad mixture of urban and rural land cover types (Fig. S1; Table S1). At each sampling site, a single 200×2 m strip transect [24] (taken to include a vertical dimension 2 m high) was defined using existing physical landmarks. Each transect was placed along a linear feature, such as a field edge or other patch boundary. In order to increase temporal resolution, subset of transects (*N* = 8), selected at random across all sites, was visited at least every 8 days, and remaining transects (*N* = 34) were visited at least every 16 days. All visits took place between 26 March and 30 June 2012. Although this sampling period may have missed foraging activity by later-founded colonies or colonies founded (via facultative bivoltinism) by newly-produced queens of the year, it should have captured the bulk of *B. hypnorum* foraging activity, since, in the study year, the corresponding time period contained 82.3% of *B. hypnorum* records for England and Wales [23]. To avoid pseudoreplication of landscape metrics, transects were kept spatially separated (the mean distance between neighbouring transects was 3100 m, range 1600–6150 m).

On each transect visit, a single observer, walking at roughly 1.5 km h^−1^, recorded all *Bombus* individuals observed on the transect (excluding socially parasitic species). The following data were collected for each bumblebee encountered: species, sex, caste (queen or worker), and forage plant visited (if any). Bumblebees were identified using [19], with *B. terrestris* and *B*. *lucorum* workers, which are difficult to distinguish in the field, being pooled (hereafter, ‘*Bombus terrestris* agg.’). When necessary, bees were temporarily caught to confirm species or sex. Transects were only surveyed in dry weather when the temperature was at least 10°C (before 1 May 2012) or 14°C (on or after 1 May 2012). The air temperature was recorded to the nearest 0.5°C with a digital thermometer; at each transect visit, values ranged from 10.0°C to 26.5°C. Since all sampling sites were on public rights of way and no endangered or protected organisms were sampled, no formal permissions were required for this work.

### Sampling of Forage Composition

At each transect visit, a 2×2 m quadrat was placed at a randomised location within 5 m of each of the points 50 m, 100 m and 150 m along the transect’s length. Abundances of all plant taxa with open flowers within the quadrat were recorded as presence/absence of open flowers in each of 25 equally-sized subdivisions of the quadrat. Species within the genera *Anchusa, Cirsium, Geranium, Malus, Papaver, Ranunculus, Rubus, Silene, Solanum, Symphytum, Taraxacum, Ulex* and *Vicia* were identified to genus level only. The quadrat was taken to be 2 m high, so that flowers on bushes within hedges and scrub vegetation were included.

### Analysis of Foraging Preferences

We quantified the foraging preference of a given *Bombus* species for a focal forage plant taxon by comparing, within transects, the proportion of its visits to that taxon (out of its total visits, with sexes and castes being pooled) with the proportion of available flowers represented by the focal plant taxon [7]. If the given *Bombus* species had no preference for the focal plant, these proportions would be the same, whereas any deviation of the proportion of visits above or below the plant’s relative abundance would indicate a foraging preference and non-preference, respectively. Preference was therefore calculated as (α – β)/β [7], where α = proportion of visits recorded by the given *Bombus* species to the focal plant taxon and β = proportion of flowering plants represented by that taxon. Where a *Bombus* species was recorded visiting a plant that was present on the transect but not recorded in the quadrats, the plant was assumed to have a relative abundance of 1%. In order to predict foraging patterns, we then calculated the quantity, ((α – β)/β) +1 (hereafter, ‘foraging preference index’). The relative abundance of a plant taxon, multiplied by the foraging preference index, predicted the expected proportion of foraging visits to the focal plant taxon. For example, if a plant taxon comprised 10% of the flowering plants and received 50% of visits from a given *Bombus* species, the foraging preference index was ((0.5–0.1)/0.1) +1 = 5. To avoid calculating foraging preference indices for plant taxa receiving very few visits, foraging preference indices were calculated for each plant taxon within the smallest set of plant taxa that collectively received more than 95% of observed visits.

Foraging preference indices for a given *Bombus* species were averaged across all transects for which the *Bombus* species co-occurred with the focal plant. We then compared the foraging preference indices of co-occurring bumblebee species groups [7]. For this purpose, given that tongue length in bumblebees affects their floral preferences, we grouped species by tongue length. Therefore, foraging preference indices were calculated for (i) all *Bombus* spp. excluding *B. hypnorum*, (ii) all short-tongued *Bombus* species excluding *B. hypnorum*, and (iii) *B. hypnorum*, and tested for significant differences between species groups using Mann-Whitney U tests. Short-tongued *Bombus* species were defined as all *Bombus* species in the data set with a proboscis length of less than 8 mm, i.e. *B. hypnorum*, *B. lapidarius, B. pratorum* and *B. terrestris* agg. *Bombus* species in the data set with proboscis lengths of more than 8 mm (*B. hortorum* and *B. pascuorum*) are hereafter referred to as long-tongued *Bombus* species. Proboscis lengths were taken from [25] and [26].

### Classification of Land Cover

Ordnance Survey (OS) MasterMap data [27]were used to generate a vector map of the area surrounding each transect using ArcGIS 10.0 [28]. Each vector map consisted of polygons defining the extent of buildings, roads, fields and other land parcels, with each polygon possessing an attribute table containing its characteristics. All polygons that at their nearest point were within 1500 m of each transect centre were included.

The attribute tables associated with the polygons on these maps were used to reclassify the land cover of given areas as urban, woodland, semi-natural or farmland using the OS MasterMap fields. Urban areas were defined as any area covered by buildings, gardens or roads; woodland as any area covered by mature trees with canopy cover of greater than 40%; semi natural as areas covered by either scattered mature trees, scrub, osiers (*Salix* plantations), heathland, wetland or rough grassland; farmland as areas under arable cultivation or managed pasture (see Supporting Information for details). A ground survey was undertaken to classify farmland further according to the crops being grown. This produced farmland classes of cereals, field bean (*Vicia faba*), intensive grass, oilseed rape (*Brassica napus*), other arable, and species-rich grass (see Supporting Information for details).

### Computation of Landscape Metrics

The vector maps generated as described above were converted to 300×300 pixel grids (hereafter, ‘rasters’) with a 100 m^2^ (i.e. 10 m×10 m) pixel size and with each pixel value representing the reclassified land cover classes at the centre of each grid cell. These rasters were clipped to three landscape scales corresponding to the circular areas lying within 250 m, 500 m and 1500 m, respectively, of the centre point of each transect. The radii of these areas were chosen to cover the range of *Bombus* worker foraging distances taken from the combined results of mass marking and genetic studies and to reflect that, while the number of estimated worker foraging distances remains relatively low, means tend to cluster between 250 m and 750 m and the highest maxima at around 1500 m [29], [30], [10], [31], [32]. Further rasters of 100 m^2^ pixel size were created with a unique pixel value for each agricultural field and a null value for all other land covers, and these were clipped to the same three landscape scales as above. The rasters were then processed using FRAGSTATS 4.0 [33] to calculate the percentage cover of each land cover class, and the total length of agricultural field edges (hereafter, ‘total edge’), within each landscape scale.

### Calculation of Forage Quality Index

For both short-tongued and long-tongued *Bombus* species over each transect visit, we used the foraging preference indices weighted by the abundances of plants to calculate a forage quality index and so quantify the attractiveness of the transect as a foraging site on the visit date (see Text S1 for details).

### Analysis of Landscape Predictors of *Bombus* Density

Poisson GLMMs using log link functions were constructed in R [34] using the lme4.0 package [35] to predict the density of each *Bombus* species (defined as counts per transect visit) from the landscape metrics, while controlling for phenological and local habitat variation. Initial data exploration indicated that species-rich grassland and field beans made up a particularly small proportion of the landscape and did not covary significantly with density of any *Bombus* species. These metrics were therefore omitted from further analyses.

Initial models included the following landscape metrics as fixed effects: total edge, percentage cover of urban, oilseed rape, semi-natural and woodland land-cover (hereafter, ‘selected landscape metrics’), with all landscape metrics being measured at the intermediate, 500-metre scale. Initial models also included the following variables as fixed covariates; date, to control for the increase in numbers of *Bombus* workers over time as colonies grow in size; and the forage quality index appropriate to the proboscis length-class of the focal *Bombus* species, to control for the attractiveness of the given transect site. Akaike’s Information Criteria (AIC) were used with the initial models to optimise the random component [36], which was allowed to be either just a random effect for site (grouping variable) or additionally to include effects for the forage quality indices (random slopes). The latter allowed for the attractiveness of a transect site to vary according to unmeasured landscape gradients, such as the quality of alternative forage sources. Alternative initial GLMMs were constructed using temperature instead of date as a fixed effect, but in every instance date produced a better fit and in no instance did this change the optimised random component.

Using the optimised random component, we fitted three maximal models for each *Bombus* species, each with the selected landscape metrics at the three spatial scales (250 m, 500 m and 1500 m) and the appropriate forage quality index and date as fixed effects. In each case an alternative model with temperature instead of date was compared; however, using date always yielded a lower AIC value and hence a better-fitting model.

The fixed effects were then refined to include only significant effects (at *P*<0.05) using a backwards stepwise sequence of single term deletion likelihood ratio tests, the resulting models being assessed with AIC values. The model with the lowest AIC compared to final models of the alternative scales is presented as the final model at the optimum scale for each *Bombus* species. Estimates of dispersion were also calculated as the Pearson residual sum of squares divided by residual degrees of freedom.

Finally, we compared the relative strength of the influence of different landscape elements on bee density across *Bombus* species and landscape scales. For this, we compared the fixed effects of the retained landscape metrics across models using the results of the likelihood ratio tests, which test the likelihood of a model including the focal landscape metric relative to the same model lacking the focal landscape metric.

## Results

### Phenology and Landscape Metrics

**Figure 1:**
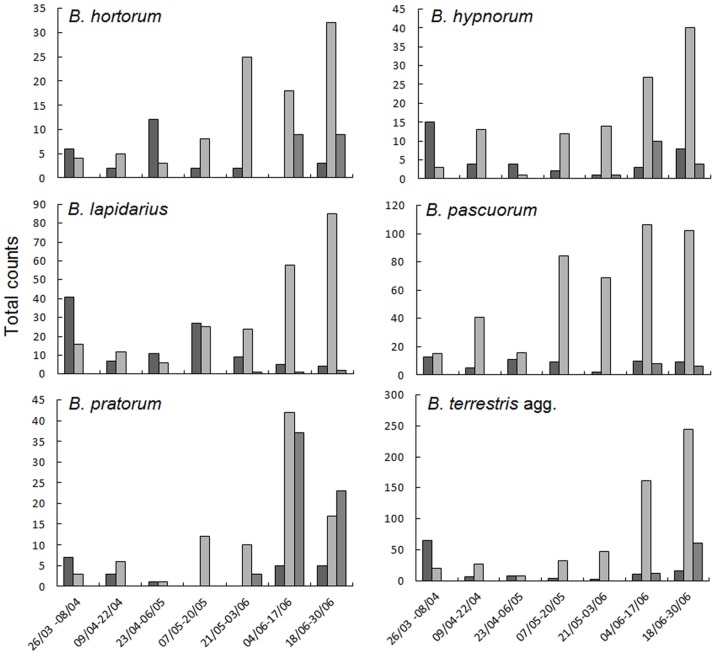
Phenologies of *Bombus* spp. across the 42 study sites (in south-eastern Norfolk, UK). Abundances shown as total counts on the transects per given two-week period (hence each period reflects approximately equal sampling effort; see ‘Materials and methods’). Dates are expressed as dd/mm in the study year, 2012. Dark grey bars, queens; pale grey bars, workers; intermediate grey bars, males.

In total, 2048 *Bombus* records were accumulated across 338 person-visits to 42 transects. Individual species contributions to the total records were as follows: *B. hortorum*, 140; *B. hypnorum*, 162; *B. lapidarius*, 334; *B. pascuorum,* 506; *B. pratorum*, 175; and *B. terrestris* agg., 731. *B. hypnorum* was recorded at 26 of the 42 transect sites and records comprised 37 queens, 110 workers and 15 males. *B. hypnorum* queens and workers were observed throughout the study period, whereas males were only observed after 28 May 2012 (Fig. 1). Our estimates of landscape metrics for the 42 sites showed that cereals represented the most frequent cover class at all scales, followed by intensive grass and urban (Table 1).

**Table 1:** Summary of landscape metrics for 42 *Bombus* transect sites in Norfolk, UK (means with range in parentheses).

Landscape metric	Information source	Scale		
		250 m	500 m	1500 m
% cover Cereals	Ground survey	28.0 (0–99.2)	29.9 (0–79.2)	35.4 (0–62.0)
% cover Intensive Grass	Ground survey	19.53 (0–93.4)	20.24 (0–90.3)	20.20 (6.2–54.6)
% cover Urban	OS MasterMap	18.8 (0–99.4)	20.1 (0.6–95.2)	17.4 (3.4–78.6)
% cover Other arable	Ground survey	9.7 (0–76. 4)	7.5 (0–60.1)	6.6 (0–22.1)
% cover Oilseed rape	Ground survey	9.3 (0–58.7)	8.2 (0–41.4)	8.8 (0–27.9)
% cover Woodland	OS MasterMap (checked via ground survey)	8.6 (0–45.9)	8.6 (0–30.6)	7.0 (0.8–18.9)
% cover Semi-natural	OS MasterMap (checked via ground survey)	3.2 (0–31.5)	2.7 (0–16.4)	2.5 (0–10.7)
% cover Species-rich grassland	Ground survey	1.9 (0–35.9)	1.2 (0–20.0)	0.5 (0–7.7)
% cover Field bean	Ground survey	0.2 (0–8.3)	0.5 (0–17.3)	0.3 (0–5.1)
Total edge (m)	OS MasterMap	3972 (0–7360)	13484 (0–25110)	32306 (2360–56820)

Scale refers to distance from the centre of the transect for which metrics were computed. Percentage cover is ranked in order of decreasing cover at the 250 m scale.

### Foraging Preferences

*B. hypnorum* was observed foraging in 140 of the total of 162 *B. hypnorum* records, distributed over 24 transect sites, during the study period. Eighteen plant taxa received 95% of all observed *Bombus* foraging visits (Table S2) and hence were used to calculate the foraging preference indices. Of these plant taxa, only nine co-occurred with *B. hypnorum* on sufficient transects to allow meaningful comparisons (taken as five or more transects).

**Figure 2:**
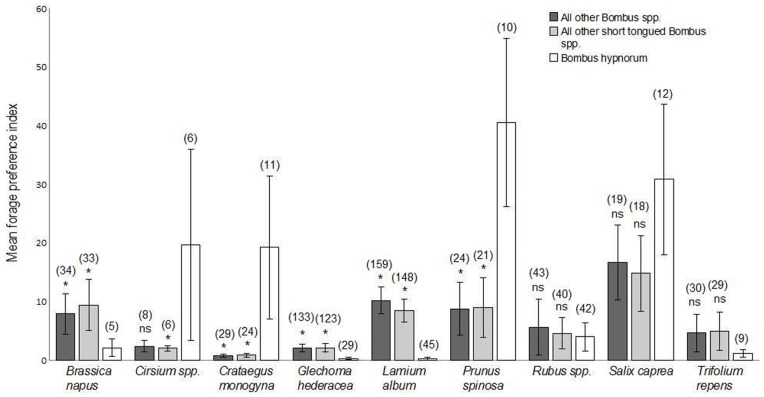
Foraging preference indices for all *Bombus* species excluding *B. hypnorum*, short-tongued *Bombus* species excluding *B. hypnorum* and *B. hypnorum*, for all plant taxa that co-occured with *B. hypnorum* on five or more transect visits. A foraging preference index >1 indicates a preference for visiting a given plant taxon (see ‘Materials and methods’). Results of Mann-Whitney U-tests between median foraging preference indices of *Bombus* species groups and *B. hypnorum*: * *P*<0.05; ns, no significant difference. Error bar is ±1 S.E. Sample sizes (number of transect-visits at which plant taxon was present and focal bee species/group was foraging) in parentheses.

Among these nine plant taxa, *B. hypnorum* exhibited the strongest foraging preferences for *Cirsium* spp., *Crataegus monogyna, Prunus spinosa* and *Salix caprea* (Fig. 2). Relative to other *Bombus* species and other short-tongued *Bombus* species, *B. hypnorum* showed a significantly higher preference for *Crataegus monogyna* and *Prunus spinosa* (Fig. 2). In addition, relative to other short-tongued *Bombus* species but not other *Bombus* species, *B. hypnorum* showed a significantly higher preference for *Cirsium* spp. (Fig. 2). *B. hypnorum* also exhibited significantly lower preferences for *Brassica napus, Glechoma hederacea* and *Lamium album* relative to other *Bombus* species and other short-tongued *Bombus* species (Fig. 2).

### Landscape Predictors of *Bombus* Density

Final models showed that in all cases except that of *B. pascuorum*, the density of each *Bombus* species was best predicted by the selected landscape metrics at the 250 m scale (Table 2; Tables S5–S9). The density of *B. pascuorum* was best predicted by the selected landscape metrics at the 1500 m scale (Table 2).

**Table 2:** Summary of final models of landscape predictors of *Bombus* density.

*Bombus* species/group	Optimum scale (m)	Fixed effects	Random effects	Dispersion estimate	AIC	ΔAIC			d.f.
						250 m	500 m	1500 m	
*B. hortorum*	250	1+ F_L_+Date+SNA	Site	1.20	338.3	–	5.9	5.9	290
*B. hypnorum*	250	1+ Date+URB +WOO+OSR	Site	0.95	267.3	–	2.1	10.8	289
*B. lapidarius*	250	1+ F_S_+SNA	Site+F_S_	0.96	483.3	–	2	0.9	249
*B. pascuorum*	1500	1+ F_L_+Date+SNA	Site+F_L_	1.21	587.7	8.9	5.1	–	247
*B. pratorum*	250	1+ F_S_+Date+URB	Site	1.48	414.8	–	1.4	2.4	290
*B. terrestris* agg.	250	1+ F_S_+Date+SNA+TE	Site+F_S_	2.87	911.0	–	4.9	2.4	247

All models are fitted to data from 338 visits to 42 transect sites. Optimum scale: landscape scale at which landscape metrics were calculated. Fixed effects: Date, date of transect-visit; F_L_, visit-specific forage quality index for long-tongued *Bombus* species; F_S_, visit-specific forage quality index for short-tongued *Bombus* species; OSR, % oilseed rape cover; SNA, % semi-natural cover; TE, total length of field edges; URB, % urban cover; WOO, % woodland cover. Random effects: Site, identity of site. Dispersion estimate: residual sum of squares divided by residual degrees of freedom. AIC: Akaike’s Information Criterion. ΔAIC: comparison to final model at alternative landscape scales. d.f: degrees of freedom, calculated as: n -1 - number of parameters.

**Figure 3:**
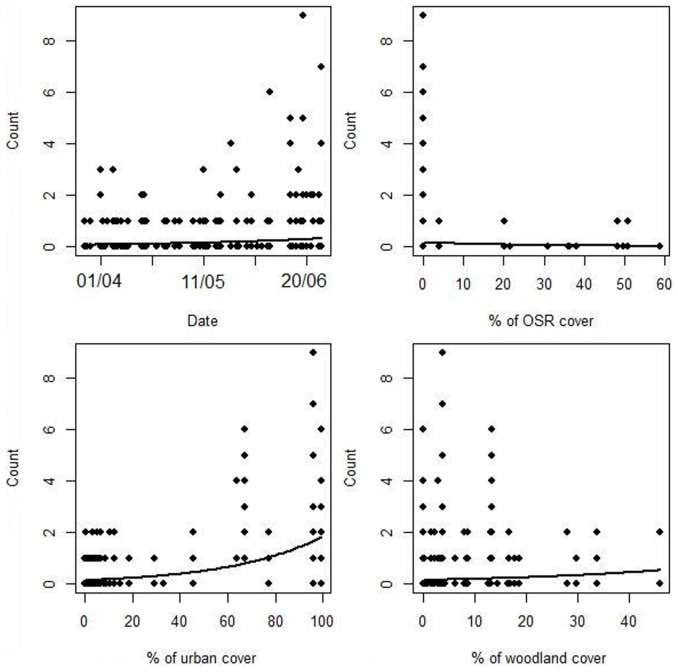
Relationship of *Bombus hypnorum* density with selected landscape metrics retained in the best-fitting final model (250 m scale:Tables 2, 3). Date expressed as dd/mm in study year (2012). Circles, counts of *B. hypnorum* observed at each transect-visit. Trend lines (plotted using LanguageR [44] from the final Poisson GLMM based on partial effects) are for illustrative purposes only; conclusions were based on results presented in Tables 2–4.

*B. hypnorum* density at the best-fitting scale (250 metres) showed a significant positive association with percentage urban cover (likelihood ratio = 38.419, *P*<0.001) and percentage woodland cover (likelihood ratio = 5.312, *P*<0.05) and a significant negative association with percentage oilseed rape cover (likelihood ratio = 8.392, *P*<0.01). The following fixed effects had no significant effect on *B. hypnorum* density: forage quality index (likelihood ratio = 2.866, *P* = 0.09), total edge (likelihood ratio = 0.044, *P* = 0.83) and percentage semi-natural cover (likelihood ratio = 1.663, *P* = 0.20) (Fig. 3; Tables 2–4). No other *Bombus* species or species group showed the combination of significant predictors of density exhibited by *B. hypnorum* (Tables 2, 4).

**Table 3:** Summary of final GLMM model of landscape predictors of *B. hypnorum* density at the optimal 250 m scale.

Fixed effect	Parameter Estimate	SE	Wald statistic	*P*
Intercept	–707.800	128.8000	–5.492	<0.001
Date	0.017	0.0031	5.477	<0.001
OSR	–0.040	0.0181	–2.190	<0.05
URB	0.026	0.0047	5.564	<0.001
WOO	0.029	0.0135	2.158	<0.05

The model is fitted to data from 338 visits to 42 transect sites. Date, date of transect-visit; OSR, % oilseed rape cover; URB,% urban cover; WOO, % woodland cover.

**Table 4:** Summary of likelihood ratios for landscape metrics at the three different scales retained as fixed effects in models predicting *Bombus* densities (data from 338 visits at 42 transect-sites).

*Bombus* species/group	Scale (m)	TE	OSR	SNA	URB	WOO
*B. hortorum*	250**	–	–	7.923	–	–
	500	–	–	–	–	–
	1500	–	–	–	–	–
*B. hypnorum*	250**	–	8.392	–	38.42	5.312
	500*	–	–	–	59.312	6.658
	1500	–	–	–	48.471	–
*B. lapidarius*	250**	–	–	5.1334	–	–
	500	–	–	–	3.1469	–
	1500*	–	4.2065	–	–	–
*B. pascuorum*	250	–	–	–	–	–
	500*	2.695	–	–	5.014	5.345
	1500**	–	–	9.618	–	–
*B. pratorum*	250**	–	–	–	3.435	–
	500*	–	–	–	–	–
	1500	–	–	1.22	–	2.797
*B. terrestris* agg.	250**	8.320	–	4.33	–	–
	500	2.932	–	–	–	–
	1500*	–	–	–	5.465	–

OSR, % oilseed rape cover; SNA, % semi-natural cover; TE, total length of field edges; URB, % urban cover; WOO, % woodland cover. ** model at landscape scale with lowest AIC; * model at landscape scale with intermediate AIC (Table 2); dash (–), fixed effect was not retained in the final model.

For most *Bombus* species, density was predicted optimally by different landscape metrics across the different scales (Table 4). For *B. hypnorum* and *B. terrestris* agg. alone, common fixed effects exhibited a significant influence across the different scales. For *B. hypnorum*, common effects were urban cover (all scales) and woodland cover (250 m and 500 m scales). For *B. terrestris* agg., the common effect was total edge (250 and 500 m scales) (Table 4). *B. pascuorum* density was predicted by a different set of predictors at the 500 m scale (urban cover and woodland cover) than at its optimal 1500 m scale (semi-natural cover) (Table 4).

Finally, the comparison of final models using likelihood tests showed that the effect of urban cover at all scales on *B. hypnorum* density was much stronger and/or more consistent (i.e. was associated with higher likelihoods) than that of any other landscape element retained in the final models for any other *Bombus* species (Table 4).

## Discussion

To elucidate the ecological factors underpinning range expansion in a naturally colonising bumblebee, we investigated the landscape predictors and foraging preferences of the Tree Bumblebee, *Bombus hypnorum*, in a representative area within its recently-colonised UK range. We found that, at the best fitting scale (250 m), *B. hypnorum* density was significantly positively associated with extent of both urban and woodland cover and significantly negatively associated with extent of oilseed rape cover. This combination of significant landscape predictors was unique to *B. hypnorum*. In addition, urban cover was associated with *B. hypnorum* density at all three scales studied and woodland cover was associated with *B. hypnorum* density at two of the three scales. Moreover, urban cover had a stronger and/or more consistent effect on *B. hypnorum* density relative to that of any other landscape elements retained in final models for other *Bombus* species. *B. hypnorum* exhibited a significantly higher foraging preference for two flowering trees, *Crataegus monogyna* and *Prunus spinosa*, relative to all other *Bombus* species studied (and hence independently of proboscis length). It also showed significantly lower preferences for the mass-flowering crop *Brassica napus* and two common, early-flowering herbaceous plants, *Glechoma hederacea* and *Lamium album*, relative to all other *Bombus* species studied (and so, again, independently of proboscis length).

The strong association shown by this study between *B. hypnorum* density and urban landscape elements (Table 4) provides quantitative support for an association previously suspected from natural-history observations alone [18], [19], [17]. The marked difference between the predictive power of urban landscape elements in explaining *B. hypnorum* density and that of any other combination of landscape element or *Bombus* species suggests the existence of quantitative ecological differences between *B. hypnorum* and other *Bombus* species. While other widespread *Bombus* species have been shown to utilise urban landscapes at high densities [37], [38], and conversely another *Bombus* species (*B. vosnesenskii*) has been shown to nest at lower densities in urbanised areas [39], this result indicates that not only does *B. hypnorum* use such landscapes in addition to the agricultural mosaic but also that urban elements represent the major component of its habitat use. This conclusion is strengthened by the fact that our final models for other *Bombus* species included significant effects for the elements that these species are known to favour in agricultural environments such as semi-natural areas and total edge [12], [38], suggesting that, if *B. hypnorum* used them widely, this would have been detected.

Our findings show that *B. hypnorum* makes use of resources offered by urban areas that are not utilised to the same extent by other *Bombus* species. Urban environments offer varied and often abundant forage in gardens, parks and waste-ground, but other species have equal access to this. Therefore a likely possibility is that *B. hypnorum* can nest at higher densities in the urban environment, relative to other environments, because of the greater availability of above-ground cavities suitable for nesting. This explanation would also be consistent with our finding that *B. hypnorum* density was significantly associated with extent of woodland cover. We note, however, that worker density is not necessarily an accurate predictor of nest density (and hence population size) in the social Hymenoptera. Hence landscape elements associated with higher *B. hypnorum* counts in our study could be facilitating either higher nest densities, or greater worker numbers per nest or a combination of these.

*B. hypnorum* may become an important crop pollinator in the UK. While it has a lower preference for *B. napus* (by far the most extensive commercial mass flowering crop in the study area) than other *Bombus* species, its early phenology and preference for *C. monogyna* and *P. spinosa* suggest it could act as a frequent pollinating visitor to spring-flowering tree-fruit crops. *P. spinosa* has several cultivated congeners, for example *Prunus avium* (sweet cherry) and *P. domestica* (plum), which typically have a flowering phenology similar to that of *P. spinosa* [40], [41]. While the economic importance of fruit crops is not as high as that of some arable mass flowering crops (e.g. *B. napus, Vicia faba*), they have been shown to be at greater risk from pollinator declines, both globally and in the European Union [5]. Overall, because *Cirsium* species, *C. monogyna* and *P. spinosa* are abundant in hedges, woodland edges and urban waste-ground, it appears that *B. hypnorum* is likely to encounter these preferred forage plants in most landscapes within the UK. *B. hypnorum* is also likely to benefit from the presence of numerous species of cultivated plants in urban environments, although such species (Table S2) could not have been identified as preferred forage species in the present study because they did not occur frequently enough across the study sites. However, it needs noting that many sources of variation were not controlled for in our calculation of foraging preference indices, including forage plant phenology and conspecific density variation.

Significant landscape predictors of bee density for *Bombus* species other than *B. hypnorum* (Table 2; Tables S5–S9) included extent of semi-natural cover (negative effect in *B. hortorum*, *B. lapidarius* and *B. terrestris* agg., positive effect in *B. pascuorum*), extent of urban cover (positive effect in *B. pratorum*) and total edge (negative effect in *B. terrestris* agg.). From previous studies identifying landscape features utilised by *Bombus* species [10], [12], [42], a positive effect of extent of semi-natural cover on *Bombus* density, as found in *B. pascuorum*, and a positive effect of total edge, might be expected. The negative effects of extent of semi-natural cover found in *B. hortorum*, *B. lapidarius* and *B. terrestris* agg., and the negative effect of total edge found in *B. terrestris* agg., are therefore unexpected. A possible explanation as regards the effects of semi-natural cover and total edge is that that a greater extent of such features caused a ‘dilution effect’ whereby densities per transect were reduced, in contrast to higher densities that can occur in resource-rich patches in a matrix of poorer-quality habitat [43]. Supporting this interpretation, in *B. pascuorum* (Table 2), in which the optimum scale was larger (1500 m), extent of semi-natural cover had a positive effect on bee density. The positive effect of extent of urban cover in *B. pratorum* accords with this species, like *B. hypnorum* but probably to a lesser degree, nesting in above-ground cavities [17].

In conclusion, this study demonstrates that, at both a landscape and within-patch scale, *B. hypnorum* and native *Bombus* species exhibit different patterns of resource utilisation. This finding could help explain the otherwise puzzlingly rapid range expansion of *B. hypnorum* within the UK. It also implies that *B. hypnorum* is unlikely to represent a competitive threat to native *Bombus* species. *B. hypnorum* may become an important pollinator of tree-fruit crops, although further research would be required to determine the efficiency with which it pollinates such crops. Because it has habitat associations differing from those of other *Bombus* species, *B. hypnorum* will also potentially provide pollination services in landscape contexts in which other species are scarce. However, in addition to the variation explained by the landscape predictors of *B. hypnorum* occurrence detected in the current study, there is clearly much unexplained variation underlying the incidence of this species (Fig. 3), which requires investigation in future work. Overall, from our current results, it appears that the quantitatively unusual combination of landscape elements favoured by *B. hypnorum* extends the range of resource utilisation patterns previously described within bumblebees. This suggests that, even in the presence of land use changes that bring about pollinator declines, management could target species with different requirements to help maintain pollination services.

## Supporting Information

Figure S1 **Sampling locations.**
(DOCX)

Table S1 **sampling locations.**
(DOCX)

Table S2 **Summary of forage plant abundances.**
(DOCX)

Table S3 **Correlation of landscape variables.**
(DOCX)

Table S4 **Summary of forage plant relative abundances across sites.**
(DOCX)

Tables S5 **Summaries of final models for other** ***Bombus*** **species densities.**
(DOCX)

Table S6 **Summaries of final models for other** ***Bombus*** **species densities.**
(DOCX)

Table S7 **Summaries of final models for other** ***Bombus*** **species densities.**
(DOCX)

Table S8 **Summaries of final models for other** ***Bombus*** **species densities.**
(DOCX)

Table S9 **Summaries of final models for other** ***Bombus*** **species densities.**
(DOCX)

Text S1 **Supporting methods detailing land cover classification and calculation of forage quality index.**
(DOCX)
