# Supplementary material for: Habitat and Forage Associations of a Naturally Colonising Insect Pollinator, the Tree Bumblebee Bombus hypnorum
Source: PLoS One. 2014 Sep 26;9(9):e107568. doi: 10.1371/journal.pone.0107568 (PMC4178030; doi:10.1371/journal.pone.0107568)

**Figure S1**. Study area in south-eastern Norfolk, U.K., showing the 42 sampling locations. Shapes denote initial (i.e. pre-land cover classification) habitat types: circles, agricultural (*N* = 16); squares, semi-natural habitat (*N* = 7); triangles, urban (*N* = 9); stars, woodland edge (*N* = 10).


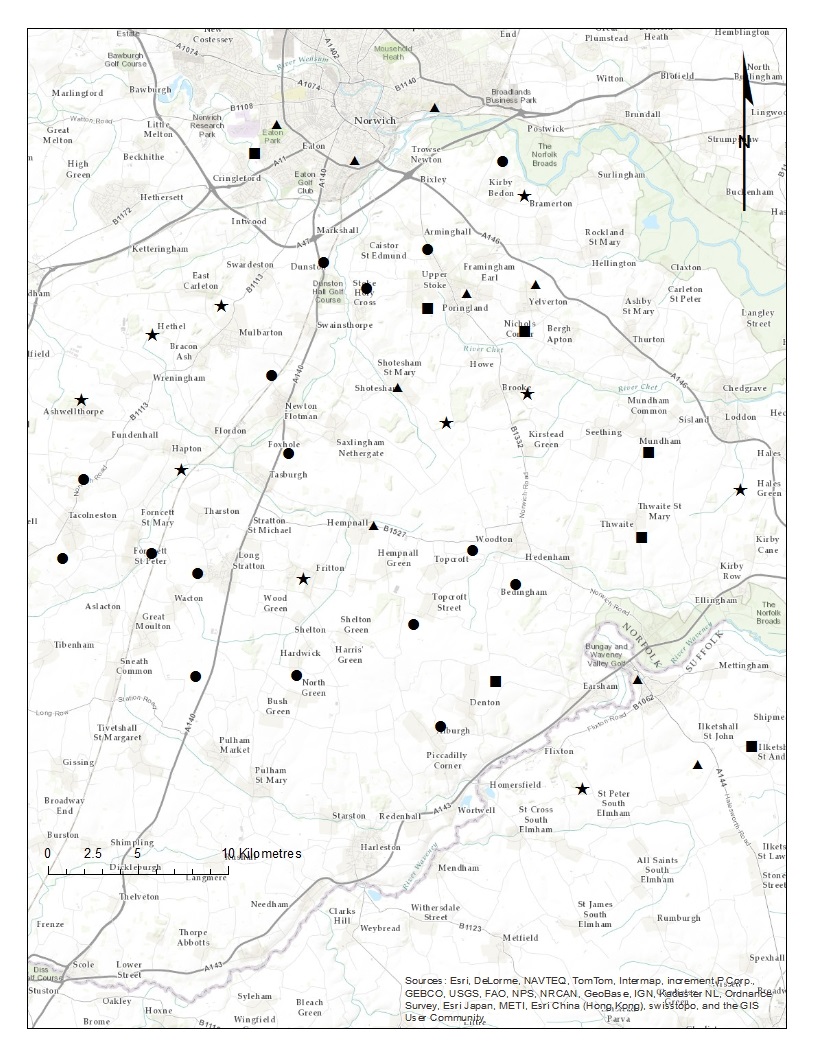

Supplement: Figure S1 — Sampling locations. (DOCX) [file pone.0107568.s001.docx]
