# Supplementary material for: Habitat and Forage Associations of a Naturally Colonising Insect Pollinator, the Tree Bumblebee Bombus hypnorum
Source: PLoS One. 2014 Sep 26;9(9):e107568. doi: 10.1371/journal.pone.0107568 (PMC4178030; doi:10.1371/journal.pone.0107568)
Supplement: Table S1 — sampling locations. (DOCX) [file pone.0107568.s002.docx]

**Table S1.** Locations of sampling sites expressed as decimal degrees using the WGS 84 coordinate system.

| Transect number | Latitude | Longitude |
| --- | --- | --- |
| 1 | 52.567133 | 1.330343 |
| 2 | 52.532698 | 1.339533 |
| 3 | 52.541441 | 1.380026 |
| 4 | 52.585124 | 1.330231 |
| 5 | 52.574603 | 1.384054 |
| 6 | 52.601736 | 1.378761 |
| 7 | 52.433840 | 1.492446 |
| 8 | 52.512255 | 1.486872 |
| 9 | 52.428337 | 1.465525 |
| 10 | 52.421029 | 1.407578 |
| 11 | 52.483150 | 1.374078 |
| 12 | 52.453744 | 1.364467 |
| 13 | 52.471104 | 1.323082 |
| 14 | 52.440134 | 1.336938 |
| 15 | 52.614506 | 1.243809 |
| 16 | 52.611944 | 1.367728 |
| 17 | 52.560371 | 1.378530 |
| 18 | 52.623198 | 1.254797 |
| 19 | 52.612224 | 1.293874 |
| 20 | 52.628202 | 1.333504 |
| 21 | 52.581180 | 1.278257 |
| 22 | 52.568196 | 1.227104 |
| 23 | 52.523505 | 1.440578 |
| 24 | 52.497524 | 1.437074 |
| 25 | 52.546827 | 1.252090 |
| 26 | 52.493649 | 1.352780 |
| 27 | 52.501339 | 1.303257 |
| 28 | 52.543274 | 1.315259 |
| 29 | 52.572435 | 1.333697 |
| 30 | 52.573385 | 1.299817 |
| 31 | 52.454407 | 1.435184 |
| 32 | 52.523207 | 1.260665 |
| 33 | 52.486678 | 1.215236 |
| 34 | 52.485226 | 1.268165 |
| 35 | 52.455648 | 1.264501 |
| 36 | 52.455223 | 1.214415 |
| 37 | 52.518373 | 1.207232 |
| 38 | 52.491191 | 1.147791 |
| 39 | 52.492701 | 1.192101 |
| 40 | 52.515192 | 1.158352 |
| 41 | 52.539506 | 1.157148 |
| 42 | 52.559242 | 1.192506 |
