# Supplementary material for: Habitat and Forage Associations of a Naturally Colonising Insect Pollinator, the Tree Bumblebee Bombus hypnorum
Source: PLoS One. 2014 Sep 26;9(9):e107568. doi: 10.1371/journal.pone.0107568 (PMC4178030; doi:10.1371/journal.pone.0107568)
Supplement: Table S2 — Summary of forage plant abundances. (DOCX) [file pone.0107568.s003.docx]

**Table S2.** Summary of flowering plant taxa present in 1014 subdivided quadrats on 338 visits to 42 transects. Plant taxa were included in the preference analysis if they were part of the smallest set of taxa that received 95% of all observed *Bombus* foraging visits. Relative abundance, percentage of total number of quadrat divisions occupied by given taxon.

| Plant taxa | Common name | Included in preference analysis? | Number of records in: |  | Relative abundance (%) |
| --- | --- | --- | --- | --- | --- |
|  |  |  | Quadrat divisions | Transect visits |  |
| *Anchusa* spp. | Alkanet | No | 20 | 4 | 0.26 |
| *Angelica archangelica* | Garden Angelica | No | 101 | 10 | 1.32 |
| *Borago officinalis* | Borage | No | 3 | 2 | 0.04 |
| *Brassica napus* | Oilseed Rape | Yes | 619 | 37 | 8.1 |
| *Cirsium spp.* | Thistle | Yes | 38 | 8 | 0.5 |
| *Cratageous monogyna* | Hawthorn | Yes | 259 | 44 | 3.39 |
| *Digitalis purpurea* | Foxglove | No | 42 | 5 | 0.55 |
| *Geranium* spp. | Geranium | No | 358 | 35 | 4.68 |
| *Geum urbanum* | Wood Avens | No | 26 | 6 | 0.34 |
| *Glechoma hederacea* | Ground Ivy | Yes | 1153 | 171 | 15.09 |
| *Heracleum sphondylium* | Common Hogweed | Yes | 90 | 21 | 1.18 |
| *Hyacinthoides non-scripta* | Bluebell | No | 5 | 2 | 0.07 |
| *Hypochaeris radicata* | Cat's Ear | Yes | 164 | 14 | 2.15 |
| *Lamium album* | White Dead Nettle | Yes | 1645 | 157 | 21.53 |
| *Lamium galeobdolon* | Yellow Archangel | No | 4 | 2 | 0.05 |
| *Lavandula* spp. | Lavender |  | 1 | 1 | 0 |
| *Lotus corniculatus* | Bird’s-foot Trefoil | Yes | 83 | 10 | 1.09 |
| *Lunaria annua* | Honesty | No | 7 | 3 | 0.09 |
| *Malus* spp. | Apple | No | 17 | 6 | 0.22 |
| *Malva parviflora* | Mallow | No | 12 | 3 | 0.16 |
| *Medicago sativa* | Lucerne | No | 40 | 3 | 0.52 |
| *Myosotis arvensis* | Forget-me-not | No | 98 | 21 | 1.28 |
| *Papaver* spp. | Poppy | No | 6 | 3 | 0.08 |
| *Phacelia tanacetifolia* | Phacelia | No | 60 | 6 | 0.79 |
| *Primula veris* | Cowslip | No | 16 | 9 | 0.21 |
| *Primula vulgaris* | Primrose | No | 5 | 2 | 0.07 |
| *Prunus spinosa* | Blackthorn | Yes | 144 | 27 | 1.88 |
| *Ranunculus* spp. | Buttercup | Yes | 475 | 58 | 6.22 |
| *Rosa canina* | Dog Rose | No | 126 | 25 | 1.65 |
| *Rubus ideaeus* | Raspberry | No | 23 | 4 | 0.3 |
| *Rubus* spp. | Bramble | Yes | 433 | 47 | 5.37 |
| *Salix caprea* | Goat Willow | Yes | 86 | 17 | 1.13 |
| *Sambucus nigra* | Elder | No | 70 | 15 | 0.92 |
| *Scabiosa* spp. | Scabious | No | 1 | 1 | 0 |
| *Silene* spp*.* | Campion | Yes | 228 | 29 | 2.98 |
| *Sinapsis arvensis* | Field Mustard | No | 22 | 9 | 0.29 |
| *Sisymbrium officinale* | Hedge Mustard | No | 33 | 9 | 0.43 |
| *Solanum* spp. | Nightshade | No | 11 | 3 | 0.14 |
| *Stachys arvensis* | Field Woundwort | Yes | 18 | 7 | 0.24 |
| *Symphytum* spp. | Comfrey | Yes | 95 | 7 | 1.24 |
| *Taraxacum* spp. | Dandelion | Yes | 80 | 25 | 1.05 |
| *Trifolium pratense* | Red Clover | Yes | 49 | 6 | 0.64 |
| *Trifolium repens* | White Clover | Yes | 417 | 30 | 5.46 |
| *Ulex spp.* | Gorse | No | 18 | 5 | 0.24 |
| *Veronica persica* | Common Field Speedwell | No | 353 | 37 | 4.62 |
| *Viburnum opulus* | Guelder Rose | No | 6 | 3 | 0.08 |
| *Vicia* spp. | Vetch | No | 101 | 14 | 1.32 |
| *Viola tricolor* | Heartsease | No | 6 | 4 | 0.08 |
