# Supplementary material for: Habitat and Forage Associations of a Naturally Colonising Insect Pollinator, the Tree Bumblebee Bombus hypnorum
Source: PLoS One. 2014 Sep 26;9(9):e107568. doi: 10.1371/journal.pone.0107568 (PMC4178030; doi:10.1371/journal.pone.0107568)
Supplement: Table S3 — Correlation of landscape variables. (DOCX) [file pone.0107568.s004.docx]

**Table S3.** Pearson product moment correlation coefficients of pairwise combinations of landscape variables for areas within 1500 m of the 42 transect sites. TE, total edge; SNA = % semi-natural cover; URB, % urban cover; CER, % cereals cover; OSR, % oil-seed rape cover; BEA, % field bean cover; OAR, % other arable cover; IGR, % intensive grass cover; SRG, % species-rich grass cover.

TE SNA URB CER OSR BEA OAR IGR SRG

TE 1.000

SNA -0.497 1.000

URB -0.694 0.340 1.000

CER 0.524 -0.472 -0.765 1.000

OSR 0.446 -0.506 -0.467 0.405 1.000

BEA 0.206 -0.265 -0.214 0.362 0.115 1.000

OAR 0.179 0.071 -0.363 0.134 -0.037 -0.047 1.000

IGR 0.155 0.169 0.021 -0.500 -0.346 -0.225 -0.115 1.000

SRG -0.056 -0.058 -0.091 -0.158 -0.031 -0.079 0.056 0.218 1.000

WOO -0.396 0.497 0.038 -0.266 -0.491 -0.225 0.141 0.166 0.261
