# Supplementary material for: Habitat and Forage Associations of a Naturally Colonising Insect Pollinator, the Tree Bumblebee Bombus hypnorum
Source: PLoS One. 2014 Sep 26;9(9):e107568. doi: 10.1371/journal.pone.0107568 (PMC4178030; doi:10.1371/journal.pone.0107568)
Supplement: Table S4 — Summary of forage plant relative abundances across sites. (DOCX) [file pone.0107568.s005.docx]

**Table S4.** Relative abundances of plant taxa used in analysis of foraging preferences of *B. hypnorum* averaged across visits to each transect. Relative abundance, proportion of total number of occupied quadrat divisions occupied by given taxon.

| Transect | Relative Abundance |  |  |  |  |  |  |  |  |
| --- | --- | --- | --- | --- | --- | --- | --- | --- | --- |
|  | *Glechoma hederacea* | *Lamium album* | *Brassica napus* | *Rubus spp.* | *Trifolium repens* | *Crataegus monogyna* | *Prunus spinosa* | *Salix caprea* | *Cirsium spp.* |
|  |  |  |  |  |  |  |  |  |  |
| 1 | 0.517 | 0.116 | 0.000 | 0.170 | 0.000 | 0.000 | 0.000 | 0.000 | 0.000 |
| 2 | 0.551 | 0.000 | 0.000 | 0.000 | 0.000 | 0.034 | 0.045 | 0.067 | 0.000 |
| 3 | 0.182 | 0.472 | 0.000 | 0.067 | 0.000 | 0.063 | 0.097 | 0.000 | 0.000 |
| 4 | 0.035 | 0.118 | 0.438 | 0.056 | 0.000 | 0.000 | 0.000 | 0.000 | 0.000 |
| 5 | 0.164 | 0.079 | 0.689 | 0.000 | 0.000 | 0.045 | 0.000 | 0.000 | 0.000 |
| 6 | 0.061 | 0.257 | 0.000 | 0.039 | 0.000 | 0.000 | 0.000 | 0.000 | 0.000 |
| 7 | 0.374 | 0.041 | 0.000 | 0.000 | 0.045 | 0.037 | 0.099 | 0.045 | 0.000 |
| 8 | 0.554 | 0.107 | 0.000 | 0.000 | 0.000 | 0.000 | 0.000 | 0.000 | 0.000 |
| 9 | 0.130 | 0.130 | 0.479 | 0.000 | 0.000 | 0.000 | 0.000 | 0.000 | 0.000 |
| 10 | 0.157 | 0.215 | 0.000 | 0.116 | 0.000 | 0.248 | 0.000 | 0.000 | 0.000 |
| 11 | 0.066 | 0.000 | 0.270 | 0.000 | 0.066 | 0.066 | 0.013 | 0.059 | 0.000 |
| 12 | 0.087 | 0.000 | 0.000 | 0.016 | 0.031 | 0.079 | 0.031 | 0.024 | 0.000 |
| 13 | 0.163 | 0.372 | 0.000 | 0.256 | 0.000 | 0.081 | 0.023 | 0.000 | 0.000 |
| 14 | 0.161 | 0.025 | 0.178 | 0.127 | 0.000 | 0.102 | 0.025 | 0.000 | 0.000 |
| 15 | 0.031 | 0.483 | 0.000 | 0.023 | 0.000 | 0.000 | 0.000 | 0.069 | 0.073 |
| 16 | 0.109 | 0.686 | 0.000 | 0.000 | 0.000 | 0.027 | 0.000 | 0.000 | 0.007 |
| 17 | 0.217 | 0.209 | 0.000 | 0.008 | 0.000 | 0.140 | 0.000 | 0.000 | 0.000 |
| 18 | 0.048 | 0.171 | 0.018 | 0.092 | 0.035 | 0.000 | 0.000 | 0.000 | 0.000 |
| 19 | 0.012 | 0.137 | 0.000 | 0.243 | 0.090 | 0.061 | 0.033 | 0.078 | 0.000 |
| 20 | 0.121 | 0.510 | 0.000 | 0.096 | 0.000 | 0.000 | 0.000 | 0.000 | 0.000 |
| 21 | 0.099 | 0.194 | 0.000 | 0.000 | 0.255 | 0.000 | 0.000 | 0.000 | 0.000 |
| 22 | 0.288 | 0.056 | 0.000 | 0.000 | 0.000 | 0.081 | 0.000 | 0.000 | 0.000 |
| 23 | 0.107 | 0.000 | 0.893 | 0.000 | 0.000 | 0.000 | 0.000 | 0.000 | 0.000 |
| 24 | 0.152 | 0.124 | 0.000 | 0.191 | 0.247 | 0.000 | 0.073 | 0.000 | 0.000 |
| 25 | 0.000 | 0.037 | 0.147 | 0.000 | 0.275 | 0.032 | 0.000 | 0.000 | 0.000 |
| 26 | 0.193 | 0.571 | 0.000 | 0.012 | 0.000 | 0.000 | 0.000 | 0.000 | 0.000 |
| 27 | 0.074 | 0.636 | 0.000 | 0.127 | 0.000 | 0.000 | 0.083 | 0.006 | 0.000 |
| 28 | 0.201 | 0.316 | 0.000 | 0.000 | 0.000 | 0.022 | 0.000 | 0.000 | 0.000 |
| 29 | 0.056 | 0.148 | 0.000 | 0.000 | 0.000 | 0.112 | 0.000 | 0.000 | 0.000 |
| 30 | 0.143 | 0.000 | 0.000 | 0.016 | 0.111 | 0.143 | 0.222 | 0.000 | 0.000 |
| 31 | 0.025 | 0.200 | 0.000 | 0.107 | 0.037 | 0.014 | 0.000 | 0.000 | 0.000 |
| 32 | 0.313 | 0.000 | 0.000 | 0.000 | 0.024 | 0.024 | 0.000 | 0.000 | 0.000 |
| 33 | 0.064 | 0.000 | 0.000 | 0.013 | 0.308 | 0.167 | 0.000 | 0.051 | 0.000 |
| 34 | 0.490 | 0.000 | 0.000 | 0.000 | 0.000 | 0.000 | 0.000 | 0.000 | 0.000 |
| 35 | 0.240 | 0.000 | 0.000 | 0.054 | 0.434 | 0.039 | 0.000 | 0.000 | 0.000 |
| 36 | 0.250 | 0.000 | 0.406 | 0.010 | 0.000 | 0.000 | 0.042 | 0.000 | 0.000 |
| 37 | 0.243 | 0.054 | 0.000 | 0.068 | 0.000 | 0.122 | 0.000 | 0.000 | 0.149 |
| 38 | 0.556 | 0.000 | 0.272 | 0.025 | 0.000 | 0.000 | 0.000 | 0.000 | 0.000 |
| 39 | 0.118 | 0.193 | 0.000 | 0.029 | 0.025 | 0.063 | 0.000 | 0.000 | 0.000 |
| 40 | 0.225 | 0.576 | 0.000 | 0.000 | 0.000 | 0.000 | 0.000 | 0.000 | 0.008 |
| 41 | 0.135 | 0.000 | 0.000 | 0.000 | 0.170 | 0.000 | 0.015 | 0.000 | 0.015 |
| 42 | 0.226 | 0.000 | 0.000 | 0.000 | 0.000 | 0.000 | 0.015 | 0.000 | 0.000 |
