# Supplementary material for: Habitat and Forage Associations of a Naturally Colonising Insect Pollinator, the Tree Bumblebee Bombus hypnorum
Source: PLoS One. 2014 Sep 26;9(9):e107568. doi: 10.1371/journal.pone.0107568 (PMC4178030; doi:10.1371/journal.pone.0107568)
Supplement: Tables S5 — Summaries of final models for other Bombus species densities. (DOCX) [file pone.0107568.s006.docx]

**Table S5**. Summary of final GLMM model of landscape predictors of *B. hortorum* at the optimal 250 m scale. The model is fitted to data from 338 visits to 42 transect sites. Date, date of transect-visit; F_L_, visit-specific forage quality index for long-tongued *Bombus* species; SNA, % semi-natural cover

| Fixed effect | Parameter Estimate | SE | Wald statistic | P value |
| --- | --- | --- | --- | --- |
| Intercept | -876.60 | 151.10 | -5.80 | < 0.001 |
| Date | 0.02 | 0.00 | 5.79 | < 0.001 |
| F_L_ | 0.02 | 0.01 | 3.80 | < 0.001 |
| SNA | -0.07 | 0.03 | 2.35 | < 0.05 |
