# Supplementary material for: Habitat and Forage Associations of a Naturally Colonising Insect Pollinator, the Tree Bumblebee Bombus hypnorum
Source: PLoS One. 2014 Sep 26;9(9):e107568. doi: 10.1371/journal.pone.0107568 (PMC4178030; doi:10.1371/journal.pone.0107568)
Supplement: Table S8 — Summaries of final models for other Bombus species densities. (DOCX) [file pone.0107568.s009.docx]

**Table S8**. Summary of final GLMM model of landscape predictors of *B. pratorum* at the optimal 250 m scale. The model is fitted to data from 338 visits to 42 transect sites. Date, date of transect-visit; F_S_, visit-specific forage quality index for short-tongued *Bombus* species; URB, % urban cover.

| Fixed effect | Parameter Estimate | SE | Wald statistic | P value |
| --- | --- | --- | --- | --- |
| Intercept | -1.19E+003 | 1.47E+002 | -8.11 | < 0.001 |
| Date | 2.90E-002 | 3.58E-003 | 8.10 | < 0.001 |
| F_S_ | 1.49E-002 | 7.15E-003 | 2.08 | < 0.05 |
| URB | 9.51E-003 | 4.27E-003 | 2.23 | < 0.05 |
