# Supplementary material for: Habitat and Forage Associations of a Naturally Colonising Insect Pollinator, the Tree Bumblebee Bombus hypnorum
Source: PLoS One. 2014 Sep 26;9(9):e107568. doi: 10.1371/journal.pone.0107568 (PMC4178030; doi:10.1371/journal.pone.0107568)
Supplement: Table S9 — Summaries of final models for other Bombus species densities. (DOCX) [file pone.0107568.s010.docx]

**Table S9**. Summary of final GLMM model of landscape predictors of *B. terrestris* agg. at the optimal 250 m scale. The model is fitted to data from 338 visits to 42 transect sites. Date, date of transect-visit; F_S_, visit-specific forage quality index for short-tongued *Bombus* species; SNA, % semi-natural cover; TE, total length of field edges.

| Fixed effect | Parameter Estimate | SE | Wald statistic | P value |
| --- | --- | --- | --- | --- |
| Intercept | -955.0000 | 77.4100 | -12.338 | < 0.001 |
| Date | 0.0233 | 0.0019 | 12.349 | < 0.001 |
| F_S_ | 0.0345 | 0.0087 | 3.970 | < 0.001 |
| SNA | -0.0258 | 0.0149 | -1.733 | < 0.05 |
| TE | -0.0002 | 0.0001 | -3.321 | < 0.01 |
