# Supplementary material for: Habitat and Forage Associations of a Naturally Colonising Insect Pollinator, the Tree Bumblebee Bombus hypnorum
Source: PLoS One. 2014 Sep 26;9(9):e107568. doi: 10.1371/journal.pone.0107568 (PMC4178030; doi:10.1371/journal.pone.0107568)
Supplement: Text S1 — Supporting methods detailing land cover classification and calculation of forage quality index. (DOCX) [file pone.0107568.s011.docx]

**Text S1. Supporting methods.**

CLASSIFICATION OF LAND COVER

The attribute tables associated with the polygons on the vector maps were used to reclassify the land cover of given areas using the OS MasterMap fields as follows. Urban areas were defined as any area covered by buildings, gardens or roads on the basis of the 'Descgroup' fields, 'buildings' and 'multi-surface'. Woodland areas were defined as any area covered in mature trees with canopy cover greater than 40% on the basis of the 'Make' field, 'natural', and the 'Descterm' fields, 'coniferous trees', 'deciduous trees' and 'mixed trees'. Semi-natural areas were defined as other wooded areas with less than 40% canopy cover on the basis of the 'Descterm' field, 'scattered'. The accuracy of these fields was checked by survey on foot on the ground for 15 polygons for both woodland and semi-natural classifications and by comparing a further 40 polygons of each type to recent aerial photographs (Bing Maps Aerial basemap layer in ArcGIS 10.0 (ESRI Inc. 2011)). Both checks showed that the classification was correct in all cases. Semi-natural was further defined on the basis of the 'Descterm' fields, 'heathland', 'osier', 'scrub' and 'wetland'. Areas of open water and quarries were excluded from further analysis. Farmland was taken to comprise all remaining polygons ('Descterm' field, 'null').

The ground survey undertaken to classify farmland further according to the crops being grown produced farmland classes of cereals, field bean (*Vicia faba*), intensive grass, oilseed rape (*Brassica napus*), other arable, and species-rich grass. Arable crops were identified where possible from a vehicle and grassland was classified by survey on foot, with intensive grass and species-rich grass having less than 25% and more than 25%, respectively, of non-grass species in the sward (excluding *Urtica* *dioica*), as estimated by two observers.

CALCULATION OF FORAGE QUALITY INDEX

The following example shows how the calculations of forage quality index were performed. Assume that at one visit to a particular transect the mean foraging preference indices for a focal set of *Bombus* species for the plant taxa present, *Glechoma hederacea, Rubus* spp., *Stachys arvensis* and *Lamium album,* were 0.33, 6.00, 0.12 and 10.23, respectively. This would have meant that these bees showed a preference for *Rubus* spp. and *Lamium album* (foraging preference index > 1), but avoided *Glechoma hederacea* and *Stachys arvensis* (foraging preference index < 1). Assume further that the abundances of the four plant taxa, recorded over the three quadrats, were: 21:14:9:20. The forage quality index of the site for short-tongued bees would then have been calculated as: (log._10_6.00 × 14) + (log._10_10.23 × 20) = 31.09. Only preferred plant taxa were included in such calculations, since the forage quality index quantified the attractiveness of the site to *Bombus* species in terms of foraging and the presence of less attractive plants was assumed not to devalue the attractiveness of the preferred ones.
